# Induction of UPR Promotes Interferon Response to Inhibit PRRSV Replication *via* PKR and NF-κB Pathway

**DOI:** 10.3389/fmicb.2021.757690

**Published:** 2021-10-12

**Authors:** Zhenbang Zhu, Panrao Liu, Lili Yuan, Zhengmin Lian, Danhe Hu, Xiaohui Yao, Xiangdong Li

**Affiliations:** ^1^Jiangsu Co-innovation Center for Prevention and Control of Important Animal Infectious Diseases and Zoonoses, College of Veterinary Medicine, Yangzhou University, Yangzhou, China; ^2^Joint International Research Laboratory of Agriculture and Agri-Product Safety, Ministry of Education, Yangzhou University, Yangzhou, China

**Keywords:** PRRSV, unfolded protein response, PKR, Nsp4, interferon response

## Abstract

Porcine reproductive and respiratory syndrome virus (PRRSV) was previously shown to induce a certain level of cellular stress during viral replication. Unfolded protein response (UPR) is a cellular stress response responsible for coping with stress and cellular survival. However, the pathway leading to the induction of UPR that may influence PRRSV replication is still unknown. Here, we found that PRRSV infection induced UPR prior to interferon response. Induction of UPR significantly enhanced the expression of interferon and interferon-related genes, thus leading to the suppression of PRRSV infection. Next, we explored the underlying mechanisms of UPR-induced antiviral response. We found that induction of UPR promoted the expression of protein kinase R (PKR), and PKR was highly correlated with the reduction of PRRSV replication. Furthermore, tunicamycin stimulation and PKR overexpression activated NF-κB and interferon response at the early stage of PRRSV infection, thus reinforcing the expression of type I interferons and proinflammatory cytokines and leading to inhibition of PRRSV. In addition, PRRSV nsp4 was shown to reduce the expression of PKR. These findings might have implications for our understandings of the host’s immune mechanism against PRRSV and a new strategy of PRRSV to evade the host antiviral immunity.

## Introduction

The endoplasmic reticulum (ER) is a cellular factory responsible for the post-translational modification and folding of cell proteins (Braakman and Hebert, 2013). Upon the stimulation by internal and external factors, the function of the ER is disturbed, accompanied by blocking protein processing and transportation. Subsequently, many unfolded or misfolded proteins accumulate in the ER, leading to a stress response through activating the unfolded protein response (UPR) pathway (Schroder and Kaufman, 2005; Karagoz et al., 2019). UPR has three important branches: inositol-requiring enzyme 1 (IRE1), the protein kinase RNA-like ER kinase (PERK), and the activating transcription factor 6 (ATF6; Ron and Walter, 2007). It is reported that viral infections, such as arbovirus, influenza virus, and HIV infections, contributed to ER stress, and the host cells might exhibit a UPR by the unscheduled accumulation of these viral proteins (Mehrbod et al., 2019). Some studies have demonstrated that the UPR was involved in innate immune mechanism and regulated viral replication by UPR-induced interferon and inflammatory cytokines. Flaviviruses infection, including dengue, Zika, West Nile, and tick-borne encephalitis viruses, induces UPR, potentiates the expression of interferon beta (IFN-β), and leads to early activation of innate antiviral responses (Carletti et al., 2019). It is reported that induction of UPR inhibits porcine reproductive and respiratory syndrome virus (PRRSV) infection, suggesting that UPR might play a role in PRRSV infection (Catanzaro and Meng, 2019). The underlying molecular mechanisms of UPR and immune response during virus infection need further verification.

Porcine reproductive and respiratory syndrome virus is one of the most important pathogens that cause huge economic losses worldwide (Neumann et al., 2005; Zhou and Yang, 2010). PRRSV is an enveloped single-stranded, positive-sense RNA virus of the order Nidovirales, family Arteriviridae, genus Porartevirus. The genome of PRRSV is about 15 kb in length, which contains at least 11 open reading frames (ORFs). The majority of the genome (around 60–70%) encodes non-structural proteins (Nsp) involved in replication (ORF1a and ORF1ab), whereas ORFs 2–7 encode structural proteins (N, M, GP2-GP5, and E) (Dokland, 2010; Fang and Snijder, 2010). PRRSV Nsps play crucial roles in viral replication, virulence, and modulation of the immune response. The previous study has demonstrated that Nsp1, Nsp2, and Nsp11 downregulate the phosphorylation of IRF3 and inhibit the activation of IFN-β promoters (Beura et al., 2010; Li et al., 2010; Wang et al., 2019). Nsp2 also functions as a deubiquitinating enzyme to degrade many antiviral proteins (Sun et al., 2010). PRRSV Nsp4 contains the 3C-like serine proteinase responsible for most Nsps processing (Li et al., 2015). Nsp4 of the highly pathogenic PRRSV (HP-PRRSV) inhibited NF-κB signaling and interfered with the expression of IRF3 and IFN-β (Huang et al., 2014). Thus, PRRSV can utilize sophisticated mechanisms to suppress the host’s immune signaling, resulting in immune evasion.

Protein kinase R (PKR) is a serine-threonine kinase that performs an important role in virus sensing, stress response, and innate immune response. PKR was first regarded as an interferon-inducible gene and activated the phosphorylation of eIF2α and downstream signaling in response to double-stranded ribonucleic acid (dsRNA) during viral infections (Lemaire et al., 2008). It is reported that PKR was activated under stress conditions, such as oxidative stress, heat shock, and ER stress (Lee et al., 2007; Chukwurah and Patel, 2018). Recent studies have shown that PKR participates in innate immune signaling in the condition of pathogens challenge. Upon virus infection, PKR can interplay with RIG-I (retinoic acid-inducible gene I) and promote the downstream signaling cascades (Zhang and Samuel, 2008). PKR also interacts with MDA5 and LGP2, the important immune sensors responsible for inducing proinflammatory cytokines and interferons (Pham et al., 2016). PKR regulates the activity of NF-κB and MAPK signaling through interacting with TRAFs and affecting the phosphorylation of IκB, resulting in the changes in the levels of proinflammatory cytokines and IFNs (Gil et al., 2004). PKR is involved in the antiviral response against many viruses. It was reported that activation of the PKR/eIF2alpha signaling inhibited replication of the Newcastle disease virus (Zhang et al., 2014). PKR suppresses Semliki Forest virus production and strongly enhances the type I interferon response (Barry et al., 2009). PKR also inhibited influenza virus and hepatitis C virus replication (Bergmann et al., 2000; Dabo and Meurs, 2012). In brief, PKR is an important protein that regulates the host’s innate immune response and viral infection. Accordingly, viruses have evolved an immune escape mechanism to counteract antiviral immunity. However, little is known about the relationship between UPR, PKR, and PRRSV.

In this study, we determined the role of PKR in UPR-induced interferon response during PRRSV infection. We demonstrated that UPR was induced following PRRSV infection, which promoted the activation of NF-κB and interferon response through the PKR pathway. The induction of UPR influenced the expression of PKR, thus suppressing the replication of PRRSV at the early stage of infection. Conversely, PRRSV Nsp4 could reduce the expression of PKR protein, which might be a new strategy for immune evasion.

## Materials and Methods

### Ethics Statement

Porcine alveolar macrophages (PAMs) in our study were isolated from lung lavage of three 6-week-old piglets approved by the Laboratory Animal Welfare and Ethics Committee of Yangzhou University. Our animal work was carried out in compliance with the Laboratory Animals—Guideline of welfare and ethics written by the General Administration of Quality Supervision, Inspection and Quarantine of the People’s Republic of China.

### Cells and Viruses

Marc-145 and HEK293T cells were cultured in Dulbecco’s modified Eagle’s medium (DMEM) (Corning, United States) with 10% fetal bovine serum (FBS) (Gibco, United States) at 37°C in 5% CO_2_. PAMs were isolated from lung lavage of three 6-week-old piglets and maintained in RPMI 1640 medium (Gibco, United States) containing 10% FBS at 37°C in 5% CO_2_. Four PRRSV strains (CHR6, SD16, XJ17-5, and Li11) were used in this study. CHR6 and Li11 were preserved in our laboratory, and SD16 and XJ17-5 were provided by Prof. Nanhua Chen from Yangzhou University. CHR6 and SD16 are classified as classical North American type PRRSV strains. XJ17-5 and Li11 strains are highly pathogenic North American type PRRSV strains. All PRRSV strains employed for the study were propagated in Marc-145 cells and titrated as 50% tissue culture infective dose (TCID_50_).

### Expression Vector Construction and Transfection

The cDNAs encoding PKR were obtained from PAM cDNA and subcloned into a pcDNA3.1-myc vector (MY1023, EK-Bioscience, China) with an N-terminal myc tag. The PRRSV nsp1-nsp12, GP2a, GP3, GP4, GP5, E, and N genes were amplified from PRRSV CHR6 strain and cloned into pmCherry-N1 Vector (632523, Takara, Japan) with an N-terminal mcherry tag. Marc-145 and HEK293T cells were seeded in six-well plates at 2 × 10^6^ cells/well; adherent cells were transfected with the indicated plasmids at a final concentration of 3,000 ng using Lipofectamine 2000 (Invitrogen, United States) according to the manufacturer’s instructions for 24 h.

### Tunicamycin Treatment

Porcine alveolar macrophages or Marc-145 cells were seeded into six-well plates (2 × 10^6^/well). After cells adhered to the cell culture dish, the cell culture medium was replaced with fresh RPMI 1640 medium containing 2% FBS. Then, cells were mock-treated or treated with tunicamycin (TM) (1 μg/ml) (Sigma, United States), a well-known inducer of the UPR, and simultaneously, cells were mock-infected or infected with PRRSV strains with the indicated multiplicity of infection (MOI) at different time points. Cells and supernatants were analyzed with Western blot, quantitative real-time reverse-transcription polymerase chain reaction (qRT-PCR), immunofluorescence, and virus titration. Three replicates were included for each treatment.

### Western Blot

Cells were harvested and lysed in a cell lysis buffer (Beyotime, China). Whole-cell lysates in each sample were quantified, and the same amounts of proteins were subjected to 12% sodium dodecyl sulfate-polyacrylamide gel electrophoresis (SDS-PAGE) and electro-transferred to polyvinyl difluoride (PVDF) membrane (Merck Millipore, United States). Membranes were blocked with 3% bovine serum albumin (BSA) (Sangon Biotech, China) in TBST (20 mM Tris-HCl PH8.0, 150 mM NaCl, 0.05% Tween 20) at room temperature for 1 h. The membranes were then incubated with indicated primary antibodies (anti-PRRSV N) (MEDIAN, Republic of Korea), -GAPDH, -p65, -PKR, -p-eIF2α, -CHOP, -p-IRF3, -ISG15, -myc, -histone 3 (Cell Signaling Technology, United States), and -mCherry (Abcam, England) at 1:1,000 at 4°C overnight. Membranes were washed with TBST buffer four times, followed by incubation of indicated secondary antibodies at a dilution of 1:5,000. GAPDH served as an internal control. Protein signals were visualized using a chemiluminescence (ECL) reagent (NCM Biotech, China). Three replicates were included for each treatment.

### Quantitative Real-Time Reverse-Transcription Polymerase Chain Reaction

The mRNA expression of PRRSV N, ATF4, CHOP, GADD34, PKR, NF-κB, and interferon related genes IFN-β, ISG56, and IFIT1was assessed by qRT-PCR. Total RNA was extracted from cells with the TRIzol reagent (TIANGEN, China). HiScript III-RT SuperMix (Vazyme, China) was used for reverse transcription according to the manufacturer’s instructions. Quantitative real-time PCR was performed using a ChamQ Universal SYBR qPCR Master Mix (Vazyme, China) on QuantStudio3 (Applied Biosystems). Reverse-transcription products were used as templates to amplify the indicated genes with primers listed in Table 1, and the data were normalized to GAPDH or HPRT1 in each sample. Relative mRNA expression was calculated using the 2^–ΔΔ*Ct*^ method, and three replicates were included for each treatment.

**TABLE 1:** List of primers for qRT-PCR.

**Primer^a^**	**Sequence (5′-3′)^b^**
ORF7 (N)-F	AAAACCAGTCCAGAGGCAAG
ORF7 (N)-R	CGGATCAGACGCACAGTATG
ATF4-F	ATGACCGAAATGAGCTTCCTG
ATF4-R	GCTGGAGAACCCATGAGGT
CHOP-F	GGAAACAGAGTGGTCATTCCC
CHOP-R	CTGCTTGAGCCGTTCATTCTC
GADD34-F	GGTGCCAACCCAGTGATGAA
GADD34-R	AGACACCTGTAGCAGGAGTGG
IFN-β-F	AGCACTGGCTGGAATGAAACCG
IFN-β-R	CTCCAGGTCATCCATCTGCCCA
ISG56-F	GCGCTGGGTATGCGATCTC
ISG56-R	CAGCCTGCCTTAGGGGAAG
PKR-F	CTCTCCCACAACGAGCACAT
PKR-R	TGTACCCTCTGGGGATGACT
NF-κB-F	TCGCTGCCAAAGAAGGACAT
NF-κB-R	AGCGTTCAGACCTTCACCGT
HPRT1-F	TGGAAAGAATGTCTTGATTGTTGAAG
HPRT1-R	ATCTTTGGATTATGCTGCTTGACC
mIFN-β-F	GCAATTGAATGGAAGGCTTGA
mIFN-β-R	CAGCGTCCTCCTTCTGGAACT
mIFIT1-F	GAAATATGAATGAAGCCCTGGA
mIFIT1-R	GACCTTGTCTCACAGAGTTCTCAA
mGAPDH-F	TGACAACAGCCTCAAGATCG
mGAPDH-R	GTCTTCTGGGTGGCAGTGAT

*^a^F, forward primer, R, a reverse primer. The letter “m” indicates that it is for a green monkey gene.*

*^b^Pig gene sequences, green monkey gene sequences, and PRRSV gene sequences were downloaded from GenBank.*

### Immunofluorescence Assay

Processed cells were fixed with paraformaldehyde (Biosharp, China) for 10 min and then permeabilized by 0.5% Triton X-100 (Solarbio, China) [diluted in phosphate-buffered saline (PBS)] for 15 min. After rinsing with PBS three times, cells were blocked with 10% BSA for 1 h at room temperature. Next, cells were incubated with an anti-PRRSV N (4A5) antibody or NF-κB p65 primary antibody overnight at 4°C. Cells were incubated with the indicated secondary antibodies for 1 h at room temperature following three washes with PBS. Finally, cells were counterstained with 4’,6-diamidino-2-phenylindole dihydrochloride (DAPI) (Beyotime, China) in PBS for an additional 5 min. All images were captured and processed using an inverted fluorescence microscope (U-HGLGPS, OLYMPUS, Japan) or a confocal laser scanning microscope (TCS SP8 STED, LEICA, Germany). Three replicates were included for each treatment.

### Detection of Interferon and Interferon-Related Genes

Marc-145 cells were mock-treated or treated with TM (100 ng/ml) and immediately mock-infected or infected with CHR6 (MOI = 1) for 0, 4, 8, 12, 24, 30, and 36 h. qRT-PCR was performed to detect the relative expressions of IFN-β, ISG56, and IFIT1 and assess the effect of TM on interferon and interferon-related genes transcription in PRRSV infected cells. Three replicates were included for each treatment.

### Antiviral Assays

Pre-treatment: Marc-145 cells were pre-treated with different concentrations of TM (0, 0.5, and 1 μg/ml) for 4 h, and then infected with PRRSV (MOI = 1) for another 24 h, PRRSV N protein level was shown using Western blot analysis. Three replicates were included for each treatment.

Co-treatment: PRRSV (MOI = 1) and various concentrations of TM (0, 0.5, and 1 μg/ml) were added to cells together for 24 h. Western blot was used to analyze the expression of PRRSV N protein. Three replicates were included for each treatment.

### Viral Binding, Entry, and Replication Assays

Empty vector (EV) and Myc-tagged PKR were transfected into Marc-145 cells for 24 h, followed by virus binding and entry assays.

For attachment assay, cells were incubated for 2 h at 4°C after PKR overexpression. Cells were infected with PRRSV at an MOI of 5 at 4°C for 1 h, in the condition that virions bind to the surface of cells but cannot enter. After rinsing with cold PBS three times, bound virions were measured by qRT-PCR. Three replicates were included for each treatment.

For entry assay, followed by PKR overexpression, cells were infected with PRRSV at an MOI of 5 for 4 h at 4°C. After binding to the cell surface, cells were washed with cold PBS three times. The cells were replaced with warm DMEM and cultured at 37°C for 2 h, allowing virions to enter the cells. Then alkaline high-salt solution [1 M NaCl and 50 mM sodium bicarbonate (pH 9.5)] was used to remove cell-surface-associated viruses (Zhao et al., 2019). Cells were collected to detect intracellular PRRSV RNA using qRT-PCR. Three replicates were included for each treatment.

For the viral replication assay, EV- and PKR-transfected Marc-145 cells were incubated with PRRSV (MOI = 1) for the indicated time (4, 8, 12, and 24 hour post infection (hpi)). The relative mRNA expression of PRRSV N was measured by qRT-PCR. Three replicates were included for each treatment.

### Luciferase Reporter Assays

Marc-145 cells cultured in 24-well plates were co-transfected with 500 ng of IFN-β, PKR expressing plasmid or EV, and 100 ng of Renilla luciferase plasmid, which served as an internal control. After 24 h post-transfection, cells were mock-infected or infected with PRRSV for another 24 h. Then, cells were harvested, and the luciferase activity was detected with a Dual-Luciferase Reporter Assay Kit (Vazyme, China) following the manufacturer’s protocol. Relative luciferase activity was normalized to that of Renilla luciferase. Three replicates were included for each treatment.

### Nuclear/Cytosol Fractionation Assays

Marc-145 cells were transfected with EV or PKR plasmid and infected PRRSV. At the time 0, 4, 8, 12, and 24 hpi, cells were harvested. Nuclear and cytoplasmic protein samples were extracted using a Nuclear and Cytoplasmic Protein Extraction Kit (Beyotime, China) following the manufacturer’s instructions. Western blots are shown to detect NF-κB p65. Histone H3 is used as an internal nuclear control, and GAPDH served as the cytoplasmic internal control. Three replicates were included for each treatment.

### Statistical Analysis

All experiments were performed with at least three independent replicates. Statistical analysis was performed using SPSS 17.0 and GraphPad Prism 5.0. Data are presented as mean ± SEM. The Student’s *t*-test and one-way ANOVA were used to analyze the data. *p* < 0.05 was considered to be significant.

## Results

### Unfolded Protein Response Is Induced Before the Interferon Response Following Porcine Reproductive and Respiratory Syndrome Virus Infection

**FIGURE 1:**
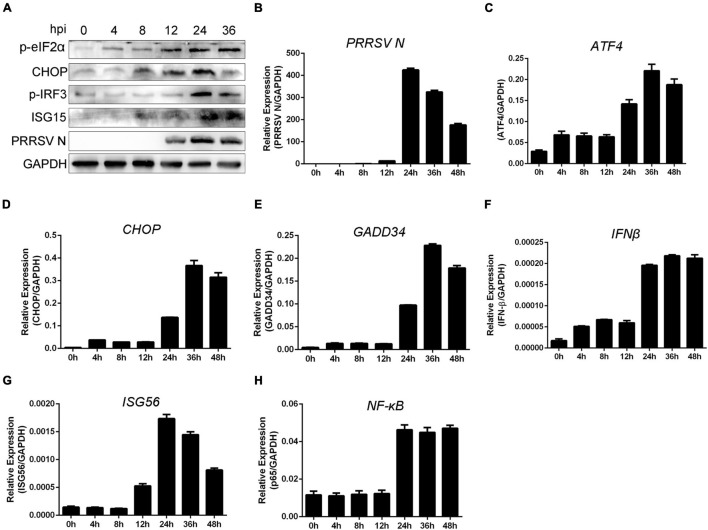
Unfolded protein response is involved in interferon response following PRRSV infection. **(A)** PAMs were infected with PRRSV (MOI = 1) for the indicated periods (0, 4, 8, 12, 24, and 36 hpi); protein levels of p-eIF2α, CHOP, p-IRF3, ISG15, and PRRSV N are shown, as detected by Western blot using the indicated antibodies. GAPDH is shown as an internal control. **(B–H)** Marc-145 cells were infected with PRRSV (MOI = 1) for 0, 4, 8, 12, 24, 36, and 48 h; total cellular RNA was extracted, and qRT-PCR was used to detect the expression of PRRSV N **(B)**, ATF4 **(C)**, CHOP **(D)**, GADD34 **(E)**, IFN-β **(F)**, ISG56 **(G)**, and NF-κB **(H)**. Data are representative of the results of three independent experiments (means ± SE).

To analyze the kinetics of UPR induction following PRRSV infection, PAMs were infected with PRRSV (MOI = 1) at the indicated time. As shown in Figure 1A, the phosphorylation of the eIF2α occurred as early as 4 hpi and was increased at the following time points. The PERK-dependent activation of CHOP was enhanced at 8 hpi. IRF3 phosphorylation was increased at 24 hpi, and the expression of ISG15 was detected at 8 hpi and increased at 24 hpi. These data suggested UPR occurred before interferon response when PAMs were infected with PRRSV. To further investigate the time order between UPR induction and interferon response, we detected the RNA expression of PRRSV N, ATF4, CHOP, GADD34, IFN-β, ISG56, and NF-κB p65 (Figures 1B–H). We found the time that the activation of UPR was consistent with interferon response. To conclude, UPR was activated at early time points following PRRSV infection, before interferon induction. Therefore, we speculated the UPR could be a prerequisite for a proper antiviral response.

### Induction of Unfolded Protein Response Inhibits Porcine Reproductive and Respiratory Syndrome Virus Replication

**FIGURE 2:**
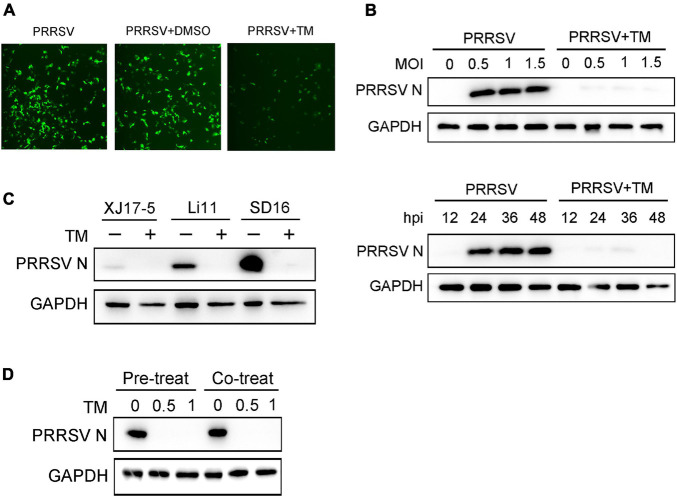
Induction of UPR effectively suppresses the replication of PRRSV. **(A)** Marc-145 cells were infected with PRRSV (MOI = 1) in the presence (1 μg/ml) or absence of TM, and DMSO is used as a control. PRRSV N protein (green) is shown using immunofluorescence analysis (Bar, 200 μm). **(B)** Western blot was used to analyze the viral N protein levels in Marc-145 cells at different PRRSV MOIs (MOI = 0, 0.5, 1, and 1.5) or different infection periods (12, 24, 36, and 48 hpi) in the presence or absence of TM (1 μg/ml). DMSO serves as a negative control. **(C)** Marc-145 cells were treated or mock-treated with TM in the presence of different strains of PRRSV (XJ17-5, Li11, and SD16); Western blot was used to detect the expression of PRRSV N. **(D)** Marc-145 cells were pre-treated with different concentrations of TM (0, 0.5, and 1 μg/ml) for 4 h, and then infected with PRRSV (MOI = 1) for another 24 h; PRRSV N protein level is shown using Western blot analysis. Meanwhile, cells were co-treated with different concentrations of TM (0, 0.5, and 1 μg/ml) and PRRSV (MOI = 1) for 24 h. Western blot was used to analyze the expression of PRRSV N. Data are representative of the results of three independent experiments.

To explore the antiviral activity of UPR against PRRSV infection, Marc-145 cells were exposed to TM, a well-known inducer of the UPR, and then infected with PRRSV CHR6 strain (MOI = 1) for 24 h. As shown in Figure 2A, when cells were treated with TM, the green fluorescence of the virus was significantly reduced, indicating that TM markedly inhibited PRRSV replication. To further confirm the inhibitory effect of UPR induction on virus infection, different MOIs of PRRSV CHR6 strain (at MOIs of 0, 0.5, 1, or 1.5) were used to infect Marc-145 cells in the absence or presence of TM (1 μg/ml). As shown in Figure 2B, TM significantly inhibited PRRSV replication regardless of the different MOI. Meanwhile, cells were exposed to TM and infected with PRRSV for the indicated time, and we found the expression of PRRSV N was reduced at 12, 24, 36, and 48 hpi. Next, we evaluated antiviral effects on different strains of PRRSV. Cells were infected with XJ17-5 strain, Li11 strain, and SD16 strain in the presence or absence of TM (1 μg/ml). As shown in Figure 2C, TM suppressed the replication of classical PRRSV strains (SD-16) and HP-PRRSV strains (XJ17-5 and Li11). We also found a significant inhibition of viral replication following the pre-treatment or co-treatment of TM in Marc-145 cells. Taken together, TM served as an inducer of UPR, which could significantly restrain the replication of PRRSV.

### Induction of Unfolded Protein Response Leads to Activation of Interferon Response During Porcine Reproductive and Respiratory Syndrome Virus Infection

**FIGURE 3:**
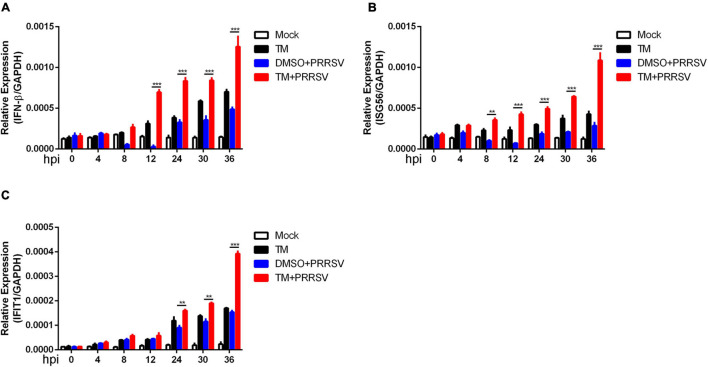
Induction of UPR accelerates the expression of interferon following PRRSV infection. Marc-145 cells were mock-infected or infected with PRRSV (MOI = 1) in the presence or absence of TM (1 μg/ml) for indicated time (0, 4, 8, 12, 24, 30, and 36 hpi). DMSO serves as a negative control. The relative expression of IFN-β **(A)**, ISG56 **(B)**, and IFIT1 **(C)** were analyzed using qRT-PCR. Data are normalized to GAPDH in each sample. Data are the results of three independent experiments (means ± SE). Significant differences are denoted by *p < 0.05, **p < 0.01, and ***p < 0.001.

Previous studies have reported that induction of UPR leads to activation of an innate antiviral response following virus infection. Marc-145 cells were mock-infected or infected with PRRSV (MOI = 1) in the presence or absence of TM (100 ng/ml) at 0, 4, 8, 12, 24, 30, and 36 hpi to determine whether UPR affects the time course of interferon response during PRRSV infection. qRT-PCR was performed to detect the expression of interferon and interferon-related genes (IFN-β, ISG56, and IFIT1). As shown in Figure 3A, when cells were infected with PRRSV alone, the expression of IFN-β decreased firstly and then increased at the late 24 hpi. Treatment of Marc-145 cells with TM alone stimulated about a five-fold increase in IFN-β mRNA after 30 h of treatment. However, upon PRRSV infection and TM treatment, IFN-β was induced as early as 12 hpi. Similarly, the expression of ISG56 was significantly increased at the early time (8 hpi) when cells were co-treated with TM and PRRSV (Figure 3B). The expression of IFIT1 was increased when cells were exposed to TM alone. Moreover, IFIT1 mRNA expression was significantly enhanced in TM- and PRRSV-cotreated cells compared to those treated with TM or PRRSV alone (Figure 3C). Collectively, induction of IFN-β, ISG56, and IFIT1 mRNA occurred much earlier following activation of UPR, suggesting induction of UPR could activate interferon response during PRRSV infection.

### Unfolded Protein Response-Induced Antiviral Signaling Following Porcine Reproductive and Respiratory Syndrome Virus Infection Potentially Requires the Protein Kinase R Pathway

**FIGURE 4:**
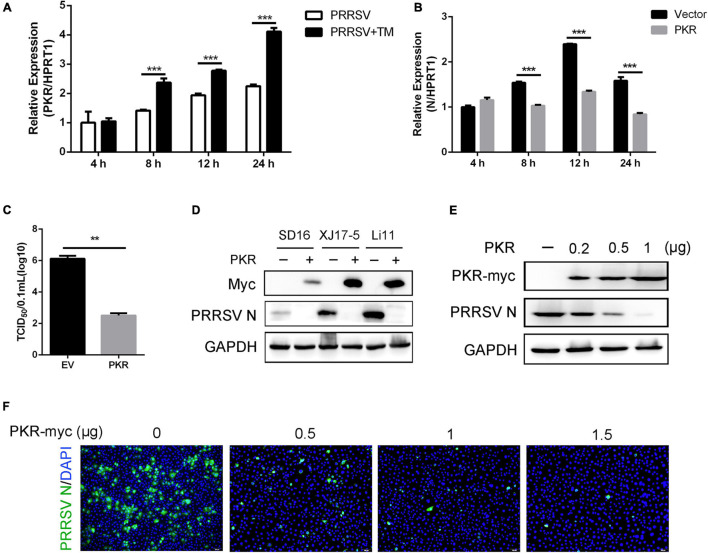
Overexpression of PKR inhibits the replication of PRRSV. **(A)** Marc-145 cells were infected with PRRSV (MOI = 1) in the presence or absence of TM (1 μg/ml) for 4, 8, 12, and 24 hpi. The mRNA expression of PKR was shown, as detected by qRT-PCR. **(B)** Marc-145 cells were transfected with pcDNA3.1-control (vector) or pcDNA3.1-PKR for 24 h and then infected with PRRSV (MOI = 1) for the indicated time (4, 8, 12, and 24 hpi). PAMs were collected, and the transcription levels of PRRSV N were shown using qRT-PCR. **(C)** Marc-145 cells were transfected with an empty vector or PKR. After overexpression for 24 h, cells were infected with PRRSV (MOI = 1) for 24 h. TCID_50_ is shown from cell supernatants. **(D)** pcDNA3.1-control plasmid and pcDNA3.1-PKR plasmid were transfected into Marc-145 cells for 24 h, and cells were infected with different strains of PRRSV (SD16, XJ17-5, and Li11, MOI = 1) for another 24 h. The expression of Myc and PRRSV N was shown, as measured by Western blot analysis. **(E,F)** Marc-145 cells were transfected with different concentrations of pcDNA3.1-PKR plasmid (0, 0.2, 0.5, 1 μg) for 24 h, and then cells were infected with PRRSV (MOI = 1). Western blot was used to detect the expression of Myc, PRRSV N, and GAPDH. GAPDH is shown as an internal control **(E)**. Immunofluorescence analysis of PRRSV N (green) expression in Marc-145 cells. Nuclei were counterstained with DAPI (blue). Bar = 200 μm **(F)**. Data are the results of three independent experiments (means ± SE). Significant differences are denoted by *p < 0.05, **p < 0.01, and ***p < 0.001.

To investigate whether PKR participated in UPR-induced antiviral response, cells were infected with PRRSV in the presence and absence of TM. Compared with PRRSV infected alone, the mRNA expression of PKR was significantly elevated in cells with TM and PRRSV cotreated at 8, 12, and 24 hpi (Figure 4A). The data suggested UPR could affect the expression of PKR. Thus, we speculated UPR was likely to stimulate interferon response to restrain PRRSV through the PKR pathway. Marc-145 cells were transfected with Myc-tagged PKR plasmids and then infected PRRSV (MOI = 1) for the indicated time to address this hypothesis. qRT-PCR was conducted to identify the mRNA expression of PRRSV N. As expected, PKR overexpression suppressed PRRSV replication as shown by decreased transcript levels of viral ORF7 at 8, 12, and 24 hpi (Figure 4B). Compared with the control vector, overexpression of PKR significantly reduced viral yields in Marc-145 cells (Figure 4C). To further investigate the antiviral effects of PKR on different PRRSV strains, Marc-145 cells were transfected with PKR and EV and infected with SD16 strain, XJ17-5 strain, and Li11 strain. As shown in Figure 4D, PKR overexpression inhibited the replication of different strains of PRRSV. In addition, we transfected different doses of PKR into Marc-145 cells and infected cells with CHR6 strain. We observed that the expression of PRRSV N was decreased in a dose-dependent manner, indicating that PKR was involved in the anti-PRRSV activity. These data demonstrated induction of UPR affected the mRNA expression of PKR, which was highly correlated with reduction of PRRSV replication.

### Protein Kinase R Suppresses Porcine Reproductive and Respiratory Syndrome Virus at the Early Stage of Replication

**FIGURE 5:**
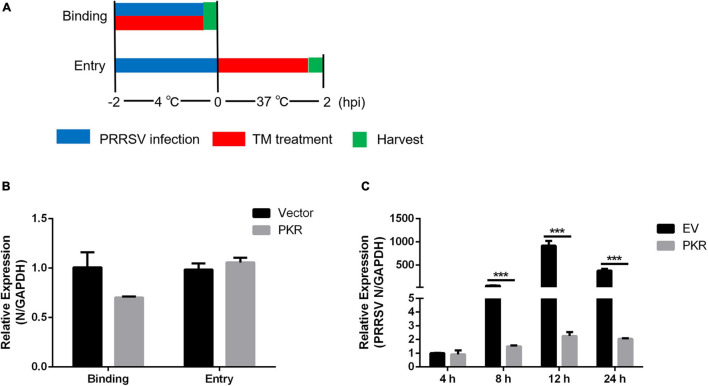
Effects of PKR on virus binding, entry, and replication. **(A)** Schematic diagram of virus binding and entry assays. **(B)** Empty vector and Myc-tagged PKR were transfected into Marc-145 cells for 24 h, followed by virus binding and entry assays. qRT-PCR was used to detect PRRSV N levels. GAPDH is shown as an internal control. **(C)** Empty vector- and PKR-transfected Marc-145 cells were incubated with PRRSV (MOI = 1) for the indicated time (4, 8, 12, and 24 hpi). The mRNA expression of PRRSV N was shown, as measured by qRT-PCR. Data are the results of three independent experiments (means ± SE). Significant differences are denoted by *p < 0.05, **p < 0.01, and ***p < 0.001.

Viral binding and entry assays were performed to identify which stage of the PRRSV life cycle was interrupted by PKR. Marc-145 cells were transfected EV or PKR plasmid for 24 h and incubated with PRRSV at an MOI of 5 at 4°C for 2 h. Cells were washed with PBS; qRT-PCR was used to detect viral attachment. For entry assay, cells were shifted to 37°C for another 2 h after virus binding to the surface, and then intracellular viral RNA was quantified using qRT-PCR (Figure 5A). As shown in Figure 5B, there was no significant difference in viral binding and entry capacity between PKR- and EV-transfected cells. These data demonstrated that PKR overexpression has little effect during the period of viral attachment and entry. Marc-145 cells were transfected with PKR and EV for 24 h and infected with PRRSV for indicated time points to investigate whether PKR overexpression affects PRRSV replication. qRT-PCR was used to quantify the PRRSV transcriptional levels. There was no significant difference in 4 hpi, which indicated PKR did not block the binding and entry of PRRSV. However, PKR overexpressing markedly decreased the expression of PRRSV N during the period of replication (from 8 to 24 hpi) compared to EV-transfected cells (Figure 5C). Collectively, PKR did not affect viral binding and entry but restrained PRRSV replication.

### PKR Promotes Activation of NF-κB and Interferon Response at the Early Stage of Porcine Reproductive and Respiratory Syndrome Virus Infection

**FIGURE 6:**
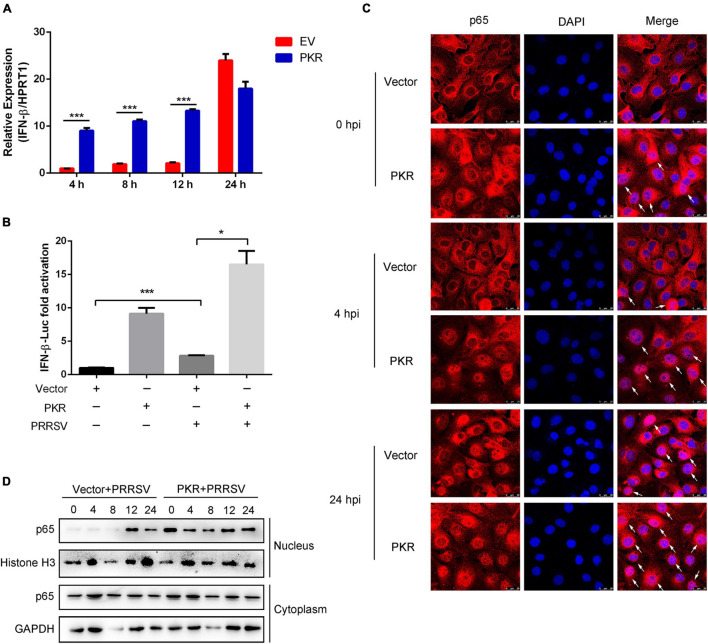
PKR promotes activation of NF-κB and interferon response. **(A)** Marc-145 cells were transfected with empty vector or Myc-tagged PKR plasmid, and then cells were infected with PRRSV (MOI = 1) for indicated post-infected time. qRT-PCR was used to measure the mRNA expression of IFN-β at 4, 8, 12, and 24 hpi. **(B)** Marc-145 cells were mock-infected or infected with PRRSV in the presence or absence of PKR overexpression. The activation of the IFN-β promoter was shown using dual-luciferase reporter assays. **(C)** Immunofluorescence analysis of p65 nuclear translocation. Marc-145 cells were transfected with empty vector or PKR plasmid and infected PRRSV for 0, 4, and 24 h. Cells were fixed and stained for NF-κB p65 followed by an Alexa fluor 555-conjugated anti-rabbit IgG (red). Nuclei were counterstained with DAPI (blue). White arrows represented p65 nuclear translocation. Bar = 25 μm. **(D)** Marc-145 cells were transfected with empty vector or PKR plasmid and infected PRRSV. At the time 0, 4, 8, 12, and 24 hpi, cells were harvested, and nuclear/cytosol fractionation assay was performed. Western blots are shown to detect NF-κB p65. Histone H3 is used as an internal nuclear control, and GAPDH serves as a cytoplasmic internal control. Data are the results of three independent experiments (means ± SE). Significant differences are denoted by *p < 0.05, **p < 0.01, and ***p < 0.001.

To demonstrate that induction of UPR stimulated interferon signaling *via* the PKR signaling pathway, Marc-145 cells were transfected with PKR and EV and then infected with PRRSV for indicated time points. Compared with EV-transfected cells, cells with PKR overexpressing significantly enhanced the expression of IFN-β at the early stage of infection. IFN-β mRNA was induced as early as 4 hpi in PKR-overexpressed cells (Figure 6A). Meanwhile, dual-luciferase reporter assay revealed that overexpressing of PKR activated the promoter activity of IFN-β in virus-infected or mock-infected cells. The activation of IFN-β was more remarkable in PKR-transfected cells than in control cells (Figure 6B). These data suggested PKR was involved in UPR-mediated interferon response.

Further study was conducted to investigate whether PKR could activate the NF-κB pathway following PRRSV infection. It is known that the nuclear translocation of p65 represents the activation of NF-κB. Using immunofluorescence analysis of the nuclear localization of p65 (red), we found that nuclear translocation of p65 occurred as early as 0 hpi in PKR-transfected cells compared to EV-transfected cells. Compared with cells treated with PRRSV alone, nuclear translocation of p65 occurred much earlier following PKR overexpression and significantly increased at 4 and 24 hpi (Figure 6C). To further determine whether PKR accelerated the activation of NF-κB following PRRSV infection, we performed a cell fractionation assay and found that nuclear accumulation of endogenous p65 occurred at 12 hpi when Marc-145 cells were treated with PRRSV alone, while it occurred much earlier (as early as 0 hpi) in PKR-overexpressed cells. Consistently, the nuclear localization of p65 was increased to a greater magnitude in cells with PKR overexpressing than controls. Taken together, PKR facilitated the activation of NF-κB and interferon response at the early stage of PRRSV infection.

### The Expression of Protein Kinase R Was Reduced in Cells That Express Porcine Reproductive and Respiratory Syndrome Virus Nsp4

**FIGURE 7:**
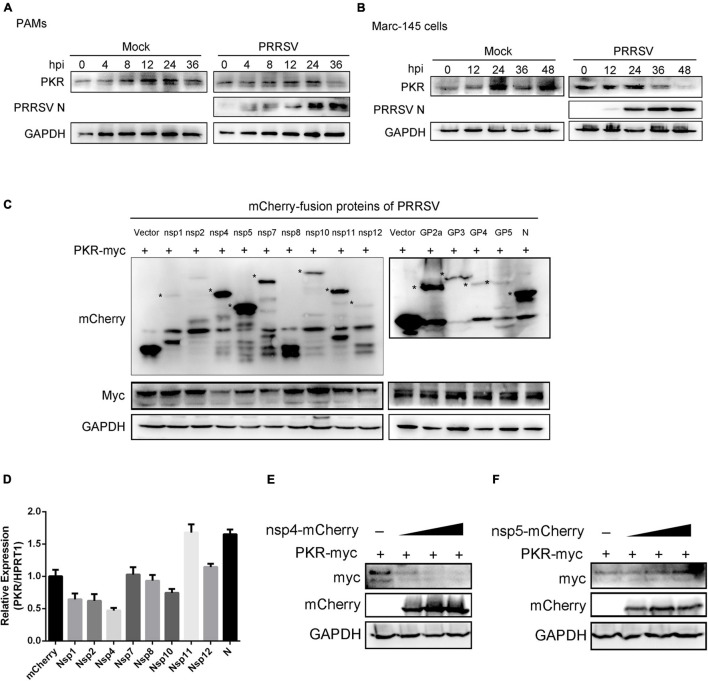
PRRSV Nsp4 inhibits the expression of PKR protein. **(A,B)** PAMs **(A)** and Marc-145 cells **(B)** were mock-infected or infected with PRRSV (MOI = 1) for 0, 4, 8, 12, 24, and 36 hpi. The protein levels of PKR and PRRSV N were shown using Western blot analysis. **(C)** HEK293T cells were co-transfected with the plasmids expressing mCherry-tagged PRRSV proteins (1.5 μg) and Myc-tagged PKR (1.5 μg). Cells were harvested at 24 h post-transfection and subjected to Western blot analysis with anti-mCherry, anti-Myc, or anti-GAPDH antibodies. **(D)** PAMs were transfected with empty plasmid or the plasmids expressing PRRSV Nsp1, Nsp2, Nsp4, Nsp7, Nsp8, Nsp10, Nsp11, Nsp12, and N, respectively. qRT-PCR was used to detect the transcript levels of PKR. Data are normalized to HPRT1 in each sample. **(E,F)** The increased doses of mCherry-tagged PRRSV Nsp4 **(E)** or Nsp5 **(F)** plasmids and Myc-tagged PKR plasmid (1.5 μg) were co-transfected into HEK293T cells for 24 h. The cell lysates were harvested and subjected to Western blot analysis with anti-Myc, anti-mCherry, or anti-GAPDH antibodies. Data are the results of three independent experiments (means ± SE). Significant differences are denoted by *p < 0.05, **p < 0.01, and ***p < 0.001.

Previous data have demonstrated that PKR affects the replication of PRRSV and interferon response. The transcript level of PKR was moderately increased when PAMs were infected with PRRSV (Figure 4A). Next, we detected the kinetics of PKR protein expression in PAMs and Marc-145 cells at various hours post-infection following PRRSV infection. Interestingly, Western blot analysis identified a marked decrease in levels of PKR protein at 36 hpi in PAMs (Figure 7A) and 36 and 48 hpi in Marc-145 cells (Figure 7B). These data suggested that some PRRSV proteins might target PKR and decrease its expression. To screen PRRSV proteins responsible for reducing PKR, mCherry-tagged viral proteins and Myc-tagged PKR were co-transfected into HEK293T cells. As shown in Figure 7C, the level of Myc-PKR protein was dramatically reduced in the cells expressing mCherry-tagged Nsp4. Similarly, eight mCherry-tagged NSPs and mCherry-tagged PRRSV-N were transfected into PAMs, respectively. It was found that the transcript level of PKR was decreased in PAMs that were expressing mCherry-tagged Nsp1, Nsp2, Nsp4, and Nsp10. Particularly, Nsp4 reduced nearly by half in comparison with control (Figure 7D). To further explore PRRSV Nsp4 and reduce the protein expression of PKR, HEK293T cells were co-transfected with increasing dose of mCherry-tagged Nsp4 and a constant dose of Myc-PKR. We observed the expression of PKR was decreased gradually, concomitant with the increase in mCherry-tagged Nsp4 (Figure 7E). However, there was no visible change when different doses of Nsp5 and Myc-PKR were co-transfected into HEK293T cells (Figure 7F). Altogether, the protein level of PKR was significantly reduced in the cells expressing Nsp4 in a dose-dependent manner, which suggested the reduction of PKR mediated by PRRSV Nsp4.

## Discussion

**FIGURE 8:**
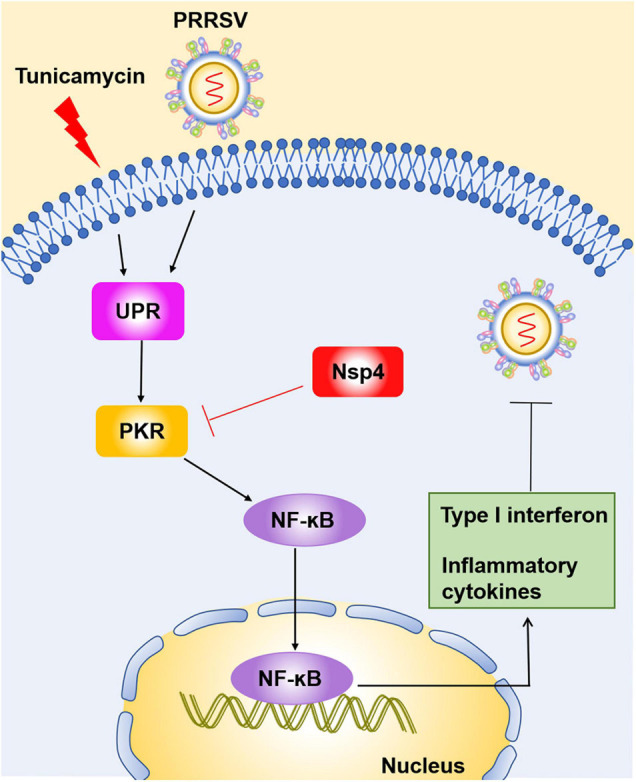
Schematic model of the inhibition of PRRSV by UPR induction. PRRSV infection and tunicamycin stimulation induce UPR. The induction of UPR affects the expression of PKR, which activates NF-κB signaling and interferon response at the early stage of PRRSV infection. Elevated type I interferons and proinflammatory cytokines are highly correlated with the reduction of PRRSV replication. Simultaneously, PRRSV Nsp4 can decrease the expression of PKR protein, indicating PRRSV evades the host immune responses *via* a new mechanism.

Porcine reproductive and respiratory syndrome virus severely impacts the global swine industry and causes significant economic losses. The relationship between PRRSV and the host’s innate immune mechanism has not yet been fully elucidated. UPR is the product of a complicated intracellular signaling pathway, which responds to the accumulation of harmful misfolded proteins in the ER and produces a protective mechanism for cells (Karagoz et al., 2019). This report identified that UPR induction activates interferon response during PRRSV infection. The mechanism is illustrated in Figure 8. First, PRRSV and TM induce the activation of UPR, which occurs before the initiation of interferon response following PRRSV infection. Second, the induction of UPR affects the expression of PKR, which possibly elicits NF-κB signaling and interferon response. The enhanced type I interferon and proinflammatory cytokines ultimately restrained PRRSV infection. Finally, PRRSV Nsp4 reduces the expression of PKR protein in a dose-dependent manner. Our data suggest PRRSV induced UPR, which promotes the host’s antiviral immune mechanism and regulates viral replication. Likewise, the PKR pathway was inclined to participate in UPR-induced interferon response. However, viral protein Nsp4 can lower the protein level of PKR. This report provides new insights into the potential interrelationship between the host and PRRSV.

The current study provides three novel insights into the UPR and PKR roles in PRRSV infection to be discussed. First, our work identified virus-induced UPR is shown to play an important role in the cell-intrinsic interferon response to PRRSV infection. Innate immunity is the first defense line to protect the host from pathogen invasion, and viral infection leads to the activation of intracellular signaling pathways that produce type I interferons and inflammatory cytokines. UPR is a stress response to unfolded and misfolded proteins in the ER in conditions of viral infection. The relevance between UPR and innate immunity has not been well studied. However, recent reports mentioned that UPR could induce an innate immune response and interferon response. Dengue, Zika, West Nile, and tick-borne encephalitis viruses activate the unfolded protein response before transcription of interferon, leading to early activation of innate antiviral responses and cell-intrinsic inhibition of viral replication (Carletti et al., 2019). Hepatitis B virus (HBV) and Kaposi’s sarcoma-associated herpesvirus (KSHV) can enhance cytokine production by activating UPR or one of its arms (Cho et al., 2015; Gilardini Montani et al., 2020). Previous studies have reported that PRRSV induces ER stress and UPR, while whether UPR affects cell-intrinsic antiviral response remains unclear. Our study first explored the time course of the activation of UPR and interferons during PRRSV infection and found that UPR occurred prior to interferon response following PRRSV infection (Figure 1). Therefore, we speculated the UPR might be a prerequisite for the cell-intrinsic antiviral response. To address this hypothesis, we induced UPR using TM and detected interferon and interferon-related genes. As expected, we found that UPR was involved in antiviral immunity during PRRSV infection. Specifically, when cells were in the presence of TM, induction of UPR accelerated the expression of interferon and cytokines, which led to the suppression of PRRSV infection (Figures 2, and 3).

Similarly, when cells were infected with PRRSV alone, UPR occurred at 4 or 8 hpi. At that time, cell-intrinsic interferon response was induced, and the expression of interferon was subsequently increased at the late time points of PRRSV infection (Figure 1A), which helps explain the time series of UPR and interferon response during PRRSV infection, suggesting that UPR response provides cellular stress signaling that contributes to optimal antiviral defense. Overall, these results suggested UPR participates in antiviral immune reactions during PRRSV infection. PRRSV infection stimulated the UPR pathway. Meanwhile, the aroused UPR restrained the replication of PRRSV *via* interferon signaling. These findings explain how UPR and interferon signaling act in concert to promote a potent response against virus infection.

Second, we verified UPR possibly affects interferon response to restrain PRRSV *via* the PKR pathway. UPR activation triggers inflammatory responses mainly through NF-κB activation, phosphorylation of JNK, and activation of the inflammasome (Chen et al., 2018). To investigate the underlying mechanisms that UPR works on immune response and PRRSV, we treated cells with TM and surprisingly found the mRNA expression of PKR was significantly increased in TM- and PRRSV-treated cells, as compared to cells with PRRSV infected alone (Figure 4A), indicating UPR could affect PKR mRNA expression. PKR, a stress-sensing protein, plays an important role in virus sensing, stress response, and innate immune response. Therefore, we speculated PKR might participate in UPR-mediated immune reactions. Our study found that overexpression of PKR significantly inhibited PRRSV replication (Figures 4, 5). Simultaneously, PKR promoted the early activation of NF-κB and the expression of IFN following PRRSV infection (Figure 6). Virus-induced UPR affects PKR expression. Besides, the antiviral effect of PKR is consistent with that of UPR, suggesting the antiviral response induced by UPR potentially depends on the PKR pathway. However, whether there is a direct relationship between UPR and PKR has not been well clarified. Furthermore, loss-of-function tests are needed to determine if UPR directly regulates PKR. In addition, PRRSV induced stress granules formation through the PERK pathway (one of the UPR branches) (Zhou et al., 2017). PKR also participates in the formation of stress granules during viral infection. Whether PKR activates antiviral signaling through cytosolic stress granules requires further investigation.

Third, it was determined that PRRSV Nsp4 could decrease the expression of PKR protein. We previously have demonstrated that PKR is likely associated with UPR-induced immune response and plays an important role in the anti-PRRSV reaction. However, many viruses utilize their proteins to counteract the activation or function of PKR. Leader protein of Theiler’s virus blocks PKR activation by preventing the interaction between PKR and viral dsRNA (Borghese et al., 2019). The 3Cpro of the foot-and-mouth disease virus induces PKR degradation through the lysosomal pathway (Li et al., 2017). Influenza A Virus NS1 inhibits activation of PKR and ensures efficient viral propagation and virulence (Bergmann et al., 2000). Likewise, some PRRSV proteins have been identified and characterized as having immune suppression roles, including IFN and NF-κB pathways inhibition (Rascon-Castelo et al., 2015). In this study, the time course of PKR expression showed that the protein levels of PKR were decreased at the late stage of PRRSV infection, indicating viral proteins could degrade PKR protein (Figures 7A,B). To investigate whether the expression of PKR protein was interrupted by PRRSV functional proteins, all the viral protein plasmids and PKR were co-transfected into HEK293T cells. The results showed that the level of Myc-PKR protein was dramatically reduced in the cells expressing Nsp4 (Figure 7C). Further data demonstrated that PRRSV Nsp4 could decrease the protein level of PKR in a dose-dependent manner (Figure 7E). PRRSV Nsp4 is a 3C-like serine protease that could cleave PRRSV Nsp3 to the Nsp12 region, attributing to its catalytic triad (His 1103, Asp 1129, and Ser 1184) (Fang and Snijder, 2010). PRRSV Nsp4 has inhibitory effects on IFN-β, NF-κB, and IRF3, thus suppressing the host’s innate immune mechanism (Chen et al., 2014). Moreover, Nsp4 can proteolytically cleave the host’s antiviral genes to antagonize antiviral activity, such as NF-κB essential modulator (NEMO), zinc finger antiviral protein (ZAP), mRNA-decapping enzyme 1a (DCP1a), and virus-induced signaling adaptor (VISA; Huang et al., 2016; Tao et al., 2018; Chen et al., 2019; Zhao et al., 2020). Considering the proteolytic enzyme activity of Nsp4, the decrease in PKR protein is possibly caused by the cleavage of Nsp4. Further investigation should be carried out to illustrate the interaction mechanism and degradation mechanism between PKR and Nsp4. Based on the above data, PKR showed an inhibitory effect on PRRSV in PKR-overexpressed cells. Meanwhile, PRRSV Nsp4 also affected the expression of PKR protein and showed antagonism to PKR proteins.

In conclusion, UPR is induced before the interferon response following PRRSV infection. The induction of UPR may affect the activation of NF-κB and IFN response through the PKR pathway, which contributes to the reduction of PRRSV replication. PRRSV Nsp4 simultaneously reduces the expression of PKR protein to escape UPR/PKR-stimulated immune response. These data provide a new understanding of host–virus interaction.

## Data Availability Statement

The original contributions presented in the study are included in the article/supplementary material, further inquiries can be directed to the corresponding author/s.

## Ethics Statement

The animal study was reviewed and approved by Laboratory Animal Welfare and Ethics Committee of Yangzhou University.

## Author Contributions

ZZ and XL conceived and designed the study, contributed to the interpretation of the data, and took part in the critical revision of the manuscript. ZZ and PL performed the experiments, analyzed the data, and drafted the manuscript. ZL, LY, DH, and XY coordinated the study. All authors have read and approved the final manuscript.

## Conflict of Interest

The authors declare that the research was conducted in the absence of any commercial or financial relationships that could be construed as a potential conflict of interest.

## Publisher’s Note

All claims expressed in this article are solely those of the authors and do not necessarily represent those of their affiliated organizations, or those of the publisher, the editors and the reviewers. Any product that may be evaluated in this article, or claim that may be made by its manufacturer, is not guaranteed or endorsed by the publisher.
